# Deficient Wnt signalling triggers striatal synaptic degeneration and impaired motor behaviour in adult mice

**DOI:** 10.1038/ncomms5992

**Published:** 2014-10-16

**Authors:** Soledad Galli, Douglas M. Lopes, Rachida Ammari, Jaakko Kopra, Sarah E. Millar, Alasdair Gibb, Patricia C. Salinas

**Affiliations:** 1 Department of Cell and Developmental Biology, University College London, London WC1E 6BT, UK; 2 Department of Neuroscience, Physiology and Pharmacology, University College London, London WC1E 6BT, UK; 3 Division of Pharmacology and Pharmacotherapy, Faculty of Pharmacy, University of Helsinki, Helsinki 00014, Finland; 4 Department of Dermatology, University of Pennsylvania, Philadelphia, Pennsylvania 19104, USA

## Abstract

Synapse degeneration is an early and invariant feature of neurodegenerative diseases. Indeed, synapse loss occurs prior to neuronal degeneration and correlates with the symptom severity of these diseases. However, the molecular mechanisms that trigger synaptic loss remain poorly understood. Here we demonstrate that deficient Wnt signalling elicits synaptic degeneration in the adult striatum. Inducible expression of the secreted Wnt antagonist Dickkopf1 (Dkk1) in adult mice (iDkk1) decreases the number of cortico-striatal glutamatergic synapses and of D1 and D2 dopamine receptor clusters. Synapse loss occurs in the absence of axon retraction or cell death. The remaining excitatory terminals contain fewer synaptic vesicles and have a reduced probability of evoked transmitter release. IDkk1 mice show impaired motor coordination and are irresponsive to amphetamine. These studies identify Wnts as key endogenous regulators of synaptic maintenance and suggest that dysfunction in Wnt signalling contributes to synaptic degeneration at early stages in neurodegenerative diseases.

## Abstract (web summary)

Synapse degeneration is an early feature of neurodegenerative diseases. Here the authors show that Wnts are endogenous regulators of synaptic maintenance and suggest that dysfunction in Wnt signalling contributes to synaptic degeneration at early stages in neurodegenerative diseases.

## Graphical abstract

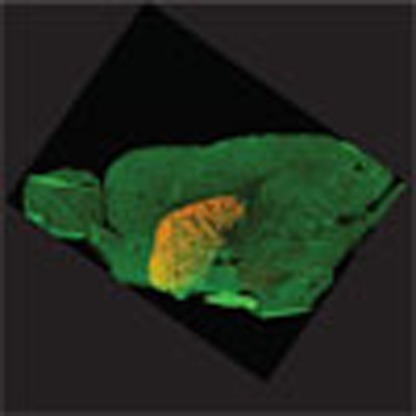

A proper balance between synapse formation, maintenance and elimination is essential for normal brain function. Although the molecular mechanisms that regulate synapse formation and growth have been well documented^1^,^2^,^3^,^4^, little is known about how synaptic stability is regulated in the adult brain. Notably, several neurodegenerative diseases exhibit early loss of synapses, with neuronal degeneration only occurring at later stages^5^,^6^,^7^,^8^. Indeed, early synapse loss often occurs concomitantly with the manifestation of the characteristic symptoms of these diseases^6^,^7^,^9^. In addition, several studies suggest that synapse loss leads to axon retraction and the subsequent death of neurons^10^,^11^. Therefore, understanding the molecular mechanisms that trigger synaptic degeneration is critical for developing therapeutic approaches to ameliorate the symptoms and prevent neuronal death in neurodegenerative diseases.

In Parkinson’s and Huntington’s disease (PD and HD), loss of synapses in the striatum results in deficits in glutamatergic and dopaminergic transmission^5^,^6^. In the striatum, the medium spiny neurons (MSNs), which represents 95% of all striatal neurons^6^,^12^, receive glutamatergic inputs from the cerebral cortex and the thalamus^12^,^13^. MSNs also receive dopaminergic inputs from the substantia nigra, which modulate glutamatergic transmission^6^,^12^. Interestingly, several proteins linked to PD and HD, such as α-synuclein (α-syn), Parkin and Huntingtin, play important roles in synaptic function^5^,^11^,^14^,^15^. Indeed, PD and HD animal models based on loss or gain of function of these genes reveal synaptic dysfunction and impaired striatal-regulated behaviour before or in the absence of neuronal death^9^,^16^,^17^,^18^,^19^,^20^. These studies support the notion that synapse loss and dysfunction occur at early stages of the disease and account for the striatal-mediated behavioural deficit. However, the molecular mechanisms that lead to synaptic degeneration in PD and HD remain poorly understood.

Recent studies suggest that Wnt signalling could play a role in synaptic maintenance in the adult brain^21^. Wnts are a family of secreted glycoproteins that promote synaptogenesis and regulate synaptic function in both vertebrates and invertebrates^1^,^2^,^3^,^4^. Indeed, we have shown that blockade of Wnt signalling by the Wnt antagonist Dickkopf1 (Dkk1) promotes shrinkage and disassembly of excitatory synapses in mature hippocampal neurons without affecting cell viability^21^. In acute hippocampal slices, amyloid-β oligomers, a key pathogenic protein in Alzheimer’s disease, increase the level of Dkk1 and promote the disassembly of synapses. Importantly, amyloid-β-induced synaptic degeneration is suppressed by blockade of Dkk1 with specific anti-Dkk1 antibodies^21^. These findings indicate that deficient endogenous Wnt signalling could contribute to synaptic degeneration in neurodegenerative diseases. However, the role of Wnts in the stability of synapses *in vivo* remains unexplored.

Here we investigated whether dysfunction of Wnt signalling *in vivo* triggers synaptic degeneration in the adult striatum, an area increasingly studied for its crucial function in motor coordination, executive function and working memory. To bypass the requirement of Wnts during embryonic and postnatal development, we established an inducible transgenic mouse model that expresses Dkk1, an effective and specific Wnt antagonist that blocks Wnt signalling by interfering with the Wnt co-receptor LRP6 (refs 22, 23). Inducible expression of Dkk1 in the adult striatum triggers the degeneration of cortico-striatal glutamatergic synapses and the disassembly of D1 and D2 dopamine receptor (D1R and D2R) clusters, without causing axon retraction or cell death. Electrophysiological recordings demonstrate an impaired cortico-striatal glutamatergic synaptic transmission. Importantly, Wnt blockade with Dkk1 results in poor motor coordination and an inability to respond to psychostimulants such as amphetamine, both classical striatal-regulated behaviours. Together, these studies identify endogenous Wnt signalling as a key regulator of synaptic maintenance/stability in the adult striatum. Our findings also suggest that deficiency in Wnt signalling could contribute to neurodegenerative diseases such as PD and HD where striatal synapses are compromised early in the disease. Moreover, we introduce the iDkk1 transgenic mice as a model system to investigate the mechanisms that control synaptic stability at early stages of midbrain-associated neurodegenerative diseases.

## Results

### Wnt signalling components are expressed in the striatum

To address the potential role of Wnt signalling in the striatum, we first evaluated the expression of Wnts, their receptors and antagonists. We examined the expression of Wnt components in the striatum of adult mice (3 months of age) and comparatively during synaptogenesis (P15 mice, the peak of synaptogenesis)^24^, by reverse transcriptase (RT)-PCR. We found that several Wnts are expressed at both stages and Wnt2, Wnt3, Wnt5a and Wnt7b are the most highly expressed Wnt genes in the adult striatum (Supplementary Fig. 1). Several Wnt receptors such as Frizzled-3 (Fz3), Fz6, Fz8 and Fz9 and the co-receptor LRP6 are expressed both in P15 and adult striatum (Supplementary Fig. 1). Wnt antagonists such as the secreted-frizzled-related proteins (Sfrps) 1–5 and Dickkopf 3 are also expressed in both stages (Supplementary Fig. 1). These results demonstrate that several Wnts, their receptors and modulators are expressed during synapse development and in the adult striatum.

### Inducible expression of Dkk1 in the dorsal striatum

To determine whether deficient Wnt signalling triggers synaptic degeneration in the adult striatum, we established a transgenic mouse model where the activity of several Wnts is blocked in the adult brain. As Wnt signalling is crucial during early embryonic patterning and morphogenesis^25^,^26^, it was essential to develop a system where Wnt signalling was unaffected during embryonic and postnatal development. To this aim, we generated an inducible transgenic line that expresses Dkk1, a specific and effective secreted Wnt antagonist^22^,^23^, upon administration of doxycycline during adulthood. In the absence of Dkk1, Wnts bind to Fz receptors and the co-receptor LRP6 resulting in stabilization of β-catenin, which translocates to the nucleus and activates gene transcription^27^. In contrast, Dkk1 blocks the binding of Wnts to Fz/LRP6, resulting in β-catenin phosphorylation and degradation by the proteasome pathway^26^,^27^. As Dkk1 is not expressed in the adult brain, inducible expression of Dkk1 in the adult provides a powerful and highly specific approach to examine the contribution of Wnt signalling to synaptic stability and function.

**Figure 1:**
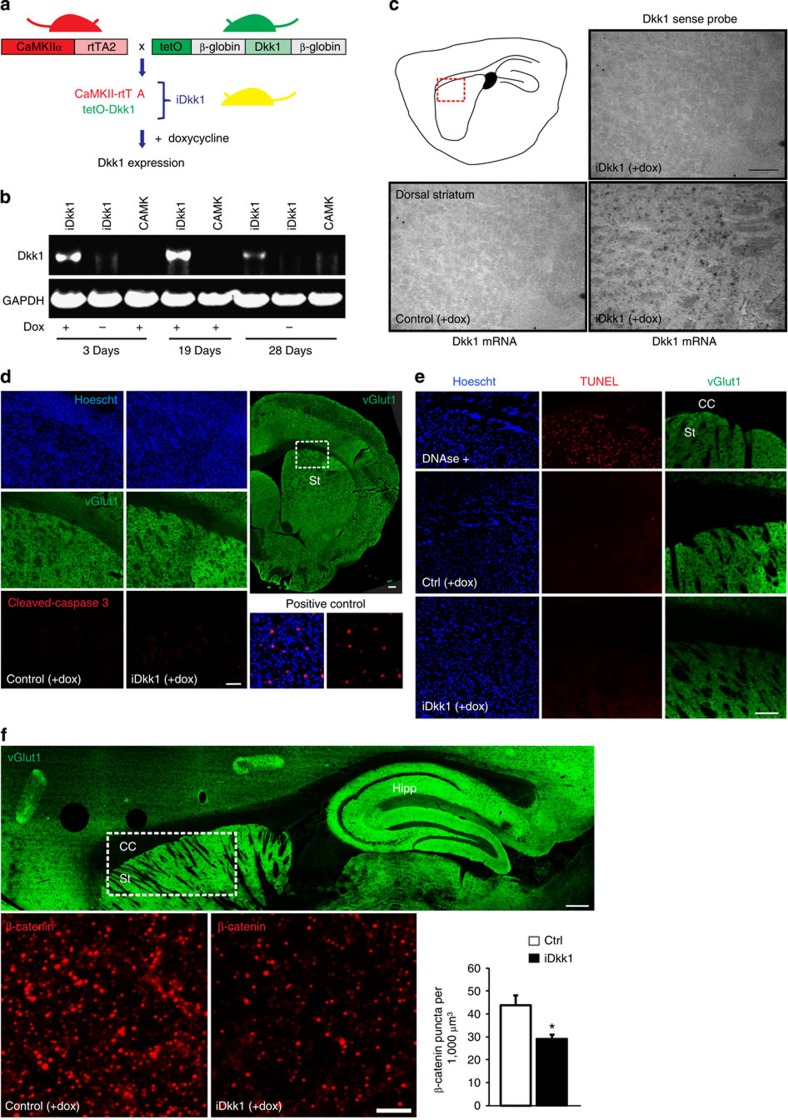
Inducible expression of Dkk1 in the striatum of adult mice blocks canonical Wnt signalling without causing cell death. (**a**) Scheme of doxycycline-induced Dkk1 expression. CaMKIIα-rtTA transgenic mice are crossed to tetO-Dkk1 transgenic mice. Double-transgenic offspring (iDkk1) are fed with doxycycline to induce Dkk1 expression. (**b**) RT–PCR showing Dkk1 expression in the striatum of adult mice after doxycycline feeding. (**c**) *In situ* hybridization of Dkk1 in mice after 14 days of doxycycline feeding. Top left, scheme of a sagittal section indicating the area shown at high magnification. Scale bar, 100 μm. (**d**) Cleaved-caspase 3 immunostaining. Top right, panoramic of a coronal slice labelled with vGlut1 depicting the striatum (St). Scale bar, 200 μm. Left, high-magnification images of area indicated in the white box. Scale bar, 100 μm. Positive control, acute cortical slices from P2 mice labelled with cleaved-caspase 3 and Hoechst. (**e**) Apoptosis evaluation by TUNEL assay. Higher-magnification images of the dorsal striatum (St) and corpus callosum (CC). Brain slices treated with DNAse I were used as positive controls. Scale bar, 100 μm. (**f**) β-catenin levels. Top, a panoramic of a sagittal section labelled with vGlut1. Scale bar, 200 μm. Below, high-magnification images obtained from the dorsal striatum (white box). Scale bar, 5 μm. Error bars represent s.e.m., *P=0.045, one-way ANOVA with replication, 9–12 images from 4 slices from each mouse and 4 mice per condition were analysed.

We used the tetracycline-inducible system, in which expression of Dkk1 is induced by administration of doxycycline during adult stages. Mice carrying the Dkk1 coding region under the control of a doxycycline-responsive promoter (tetO)^28^ were crossed to mice carrying the doxycycline-controlled transactivator (rtTA) downstream of the CaMKIIα promoter^29^. Dkk1 expression is induced in double-transgenic animals (iDkk1 hereafter) by addition of doxycycline into the diet when animals reach adulthood (Fig. 1a). CaMKIIα is highly expressed in striatal MSNs^30^,^31^ and indeed, the CaMKIIα-rtTA system can effectively drive gene expression under the tetO system in the striatum^29^,^32^,^33^.

To induce expression of Dkk1, iDkk1 mice (3–6 months old, adult mice hereafter) were fed with doxycycline for different periods of time. In all experiments, a combination of wild-type (wt) or single-transgenic mice (CaMKII-rtTA or tetO-Dkk1) were fed with doxycycline and used as controls. Expression of Dkk1 is detected by RT-PCR only in iDkk1 mice fed with doxycycline, but not in single-transgenic mice fed with doxycycline, or in iDkk1 mice not fed with doxycycline (Fig. 1b). Dkk1 expression is detected after 3 days of doxycycline exposure and is sustained as long as animals are fed with doxycycline (Fig. 1b). We next examined the expression of Dkk1 in the striatum by *in situ* hybridization. Indeed, Dkk1 messenger RNAs are detected in striatal cells of iDkk1 mice but not in single-transgenic littermates after 14 days of feeding with doxycycline, or in iDkk1 mice using a Dkk1 sense probe (Fig. 1c). The pattern is consistent with the expression driven by CaMKIIα promoter in MSNs^29^. Given that Dkk1 specifically blocks canonical Wnt signalling, these transgenic mice provide a model system to investigate the role of this signalling pathway in synaptic stability and function in the adult brain.

### Dkk1 blocks canonical Wnt signalling without cell death

IDkk1 mice not exposed to doxycycline develop normally and have no overt sensory motor defects based on their ability to move normally. To study the impact of Wnt blockade in the adult brain, Dkk1 expression was induced for 2 weeks (hereafter), as this period is required for full induction of the CaMKII-rtTA/tetO system with doxycycline^33^. By visual inspection, we found that induction of Dkk1 expression does not affect the overall morphology of the brain. To further characterize these mice, we examined possible changes in cell viability as several studies have shown that increased Dkk1 levels are correlated with increased neuronal death in models of epilepsy, ischaemia and Alzheimer’s disease^34^,^35^,^36^. However, the level of cell death is not affected in the adult striatum of iDkk1 mice, as determined by the level of activated caspase 3 and by the terminal deoxynucleotidyl transferase dUTP nick end labelling (TUNEL) assay (Fig. 1d,e). As CaMKII-rtTA can also drive the expression of Dkk1 in the somatosensory cortex^29^, we evaluated cell death in cortical areas by the TUNEL assay and by labelling with activated caspase 3. However, we observed no changes in cell death in the somatosensory cortex of iDkk1 mice when compared with controls (Supplementary Fig. 2). In addition, no difference in the total number of striatal and cortical neurons, labelled with the specific marker NeuN, or in the total number of striatal and cortical cells (labelled with the nuclear marker Hoechst) was observed in iDkk1 mice when compared with controls (Supplementary Fig. 3). Together, these findings demonstrate that induction of Dkk1 expression for 2 weeks does not affect cell viability or the overall structure of the brain.

Dkk1 is a well-established Wnt antagonist that specifically blocks the canonical Wnt pathway^22^,^23^, resulting in increased β-catenin phosphorylation and degradation^26^,^27^. We therefore examined the levels of β-catenin in iDkk1 mice. We found that some β-catenin puncta colocalize with the pre- or postsynaptic terminal of excitatory synapses, labelled by the specific presynaptic marker vGlut1 or the postsynaptic marker PSD95 (~10% colocalization), and also with nuclei (~3% colocalization). However, most of the β-catenin puncta are located to other structures (Supplementary Fig. 4). Thus, β-catenin puncta are present in different cell compartments in the striatum. As expected, when we analysed the iDkk1 mice, we found a significant decrease of β-catenin puncta (~30% decrease, Fig. 1f). Given the low level of colocalization of β-catenin to synapses, these findings indicate that the decrease in β-catenin puncta mainly occurs outside synapses. These findings show that canonical Wnt signalling is compromised in iDkk1 mice.

### Dkk1 triggers degeneration of cortico-striatal synapses

Our previous studies using mature hippocampal neurons demonstrated that blockade of Wnt signalling with Dkk1 induces the rapid disassembly of synapses in mature cultured hippocampal neurons^21^. Therefore, we proposed that Wnt signalling plays a role in synapse maintenance in the adult brain. Here, we examined whether deficient Wnt signalling in the adult striatum leads to synaptic degeneration.

**Figure 2:**
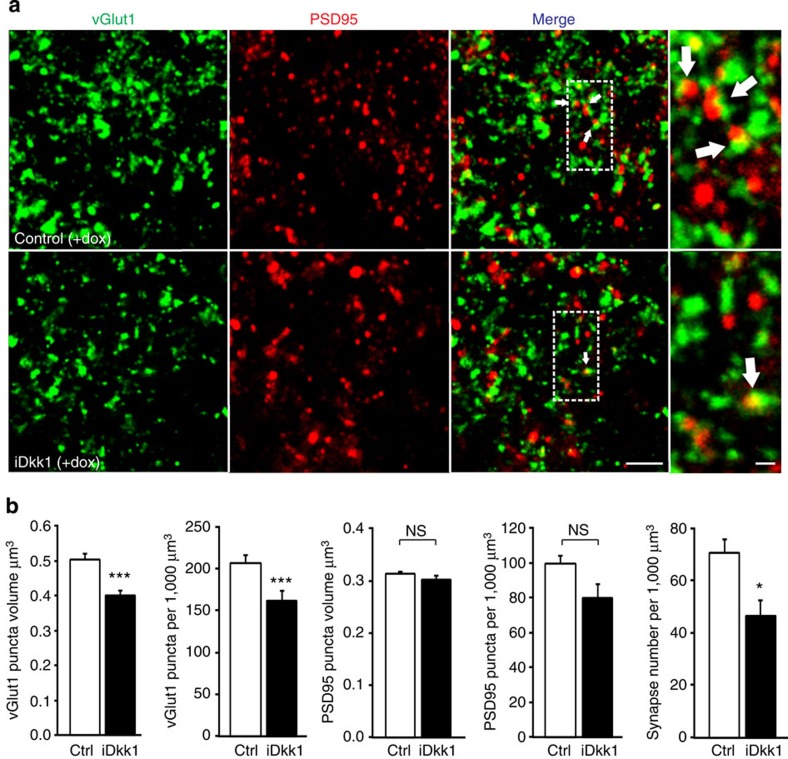
Blockade of Wnt signalling triggers degeneration of cortico-striatal glutamatergic synapses in the adult striatum. (**a**) Maximum projection confocal images of a striatal section showing presynaptic vGlut1, postsynaptic PSD95 and cortico-striatal synapses (colocalization of vGlut1 and PSD95, white arrows). (**b**) Quantification. *P<0.05, ***P<0.001, 1-way ANOVA with replication, *n*=22 images from 3 slices per control mice or *n*=16 images from 3 slices per iDkk1 mice (4 control and 5 iDkk1 mice were used). Scale bar, 5 μm. NS, not significant.

Striatal MSNs receive glutamatergic input from cortex and thalamus^12^, which can be distinguished by the differential expression of vGlut1 or vGlut2 in the presynaptic terminal of cortical or thalamic inputs, respectively^13^,^37^. We first assessed the effect on cortico-striatal excitatory synapses by evaluating the colocalization of vGlut1 and the postsynaptic marker PSD95. Although we measured a decrease in the number of PSD95 puncta following Dkk1 induction, this was not statistically significant (Fig. 2). However, Dkk1 expression significantly decreases the volume and number of vGlut1 puncta, as well as the colocalization of vGlut1 with PSD95 (~30% decrease, Fig. 2). Thus, blockade of Wnt signalling in the adult striatum results in degeneration of cortico-striatal glutamatergic synapses.

**Figure 3:**
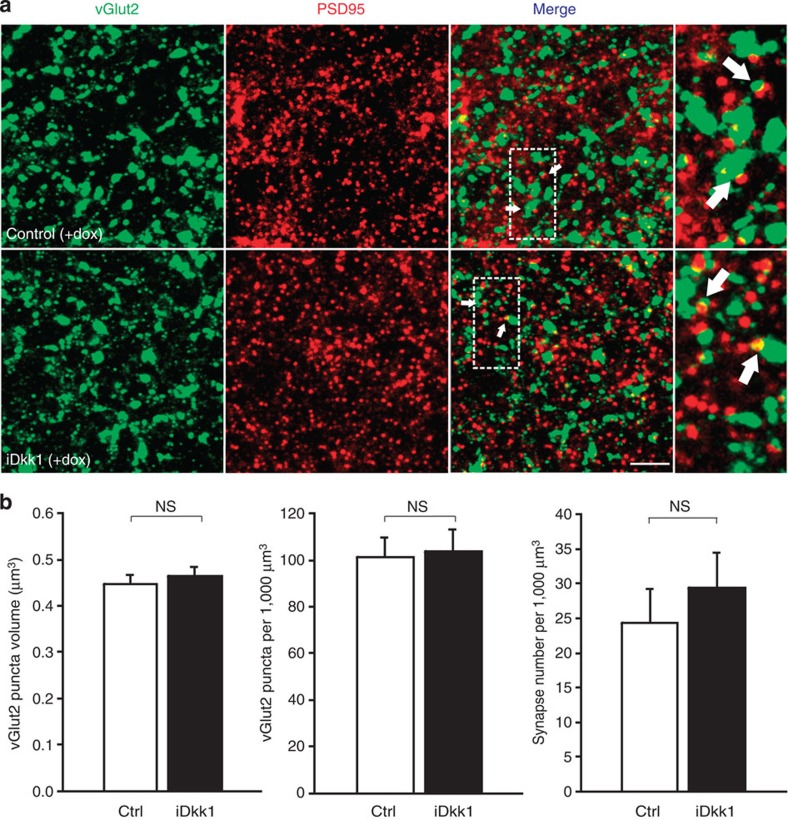
Blockade of Wnt signalling in the adult striatum does not induce disassembly of thalamo-striatal glutamatergic synapses in the dorsal striatum. (**a**) Maximum projection confocal images of striatal sections showing presynaptic vGlut2, postsynaptic PSD95 and thalamo-cortical synapses, evaluated as the colocalization of vGlut2 and PSD95 (white arrows). (**b**) Quantification. One-way ANOVA with replication, 9–12 images were taken from 4 slices from each mouse, and 4 mice were analysed per condition. Scale bar, 5 μm. Error bars represent s.e.m. NS, not significant.

We next evaluated whether blockade of Wnt signalling affects thalamo-striatal glutamatergic synapses by examining the colocalization of vGlut2 and PSD95. In contrast to vGlut1, induction of Dkk1 in the striatum does not significantly affect the volume or number of vGlut2 puncta or the number of colocalized vGlut2- and PSD95-labelled synapses (Fig. 3). Together these findings demonstrate that Dkk1 specifically affects the maintenance of cortico- but not thalamo-striatal synapses in the adult striatum.

To further evaluate the effect of Dkk1 on glutamatergic synapses, we analysed synapses at the ultrastructural level. Using electron microscopy (EM) we observed a decrease in the number of asymmetric synapses, that is, glutamatergic synapses, in the striatum of adult iDkk1 mice (~17% decrease, Supplementary Fig. 5). This decrease is smaller to that observed by the colocalization of vGlut1 with PSD95 (30% decrease of cortico-striatal glutamatergic synapses). This difference could be explained by the fact that Dkk1 preferentially affects cortico-striatal but not thalamo-striatal glutamatergic synapses, which represent ~50% of glutamatergic striatal synapses^13^. Taken together, these results demonstrate that endogenous Wnt signalling is crucial for the maintenance of specific glutamatergic synapses in the striatum.

**Figure 4:**
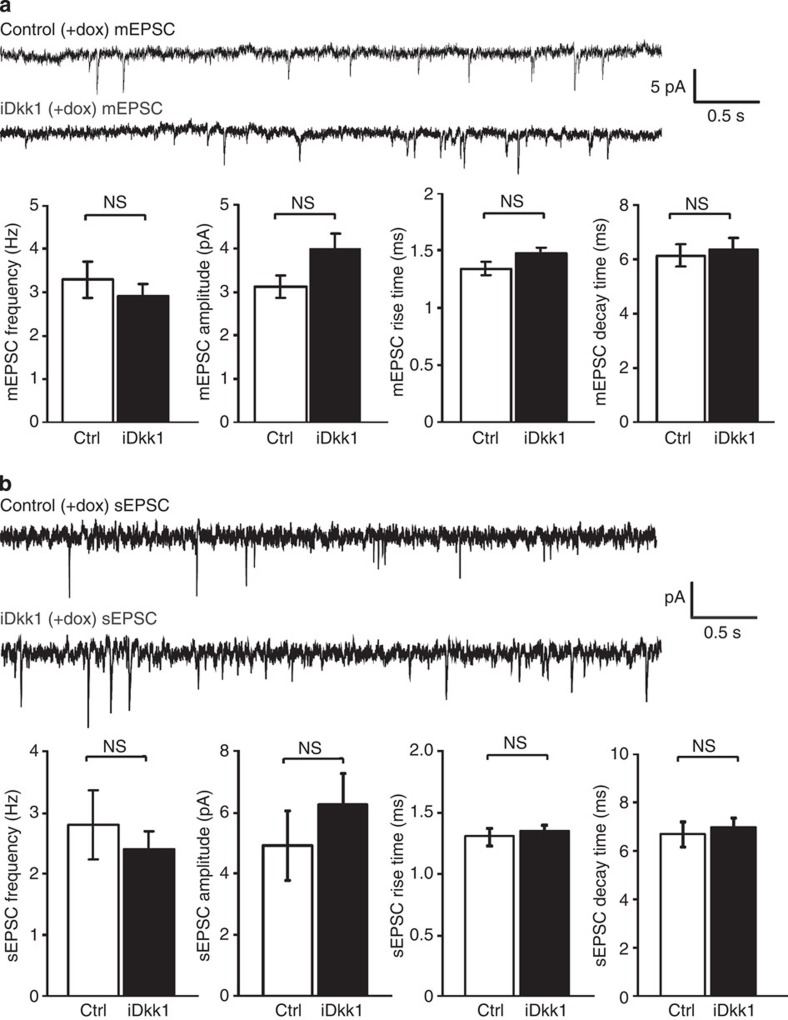
Dkk1 expression in adult striatum does not affect mEPSCs and sEPSCs. Representative electrophysiological recordings of mEPSC (**a**) and sEPSC (**b**) of MSN neurons held at Vm=−70 mV. Graphs illustrate the amplitude and kinetic properties of mEPSCs and sEPSCs. Error bars represent s.e.m. Student’s *t*-test, *n*=20 and *n*=27 neurons were recorded from control and iDkk1 mice for mEPSCs, respectively, and *n*=16 and *n*=17 neurons from control and iDkk1 mice for sEPSCs, respectively. NS, not significant.

To investigate functional changes in excitatory synaptic function, we recorded miniature excitatory postsynaptic currents (mEPSCs) and spontaneous postsynaptic currents (sEPSCs) in striatal MSNs. Induction of Dkk1 expression does not affect the frequency or amplitude of mEPSCs or sEPSCs (Fig. 4). These results are unexpected, given the overall decrease in the glutamatergic input in adult iDkk1 mice (~17% as detected by EM, Supplementary Fig. 5). Our interpretation is that the overall decrease in the number of excitatory synapses is not sufficient to significantly change mEPSCs and sEPSCs, although there is a significant decrease in the number of excitatory cortico-striatal synapses.

### Dkk1 impairs presynaptic structure and function

**Figure 5:**
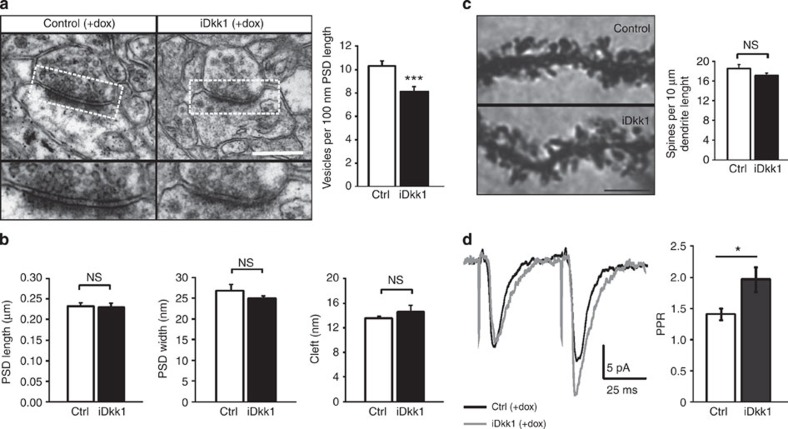
Blockade of Wnt signalling decreases the number of vesicles in the adult striatum. (**a**) Electron micrographs (top) and quantification (bottom) of number of vesicles in asymmetric synapses from the dorsal striatum. Scale bar, 1 μm. ***P<0.001, one-way ANOVA with replication, *n*=17–25 synapses for each control and *n*=15–19 synapses for each iDkk1 mice, 4 mice for each condition. (**b**) PSD length, width and synaptic cleft were analysed in the electron micrographs of asymmetric synapses described in Supplementary Fig. 5. Kruskal–Wallis ANOVA, *n*>140 synapses obtained from 4 mice for each condition. (**c**) Left, sections of Golgi-stained dendrites of medium spiny neurons in the dorsal striatum. Right, quantification of spine density. Kruskal–Wallis ANOVA, 9–50 dendrite stretches obtained from each mouse and 4 mice for each condition. Scale bar, 5 μm. (**d**) Top, representative averaged traces of evoked excitatory postsynaptic currents (eEPSC) obtained from stimulation of cortical afferents onto one MSN held at Vm=−70 mV. Bottom, paired-pulse ratio (PPR), *P=0.016, unpaired *t-*test, *n*=14 cells for control and *n*=18 cells for iDkk1 mice. Error bars represent s.e.m. NS, not significant.

Our EM analyses and colocalization of pre- and postsynaptic markers demonstrate that a number of excitatory synapses are refractory to dysfunction in Wnt signalling. To investigate the potential effect of Dkk1 on the remaining synapses, we examined the pre- and postsynaptic ultrastructure. We observed that blockade of Wnt signalling in iDkk1 mice decreases the number of vesicles at the active zone (Fig. 5a). In contrast, no significant changes in the PSD length and width, or synaptic cleft width are observed (Fig. 5b), consistent with our confocal immunofluorescence analyses where the number and size of PSD95-labelled puncta are unaffected in iDkk1 mice (Fig. 2). No changes in the number of dendritic spines in MSNs of adult iDkk1 mice are observed by Golgi staining (Fig. 5c). Together these findings demonstrate that Dkk1 affects predominantly the presynaptic side of excitatory synapses.

In light of these findings, we investigated whether transmitter release was affected by recording EPSCs in MSNs. Evoked responses were obtained by stimulation of cortico-striatal afferents using a paired-pulse stimulation protocol. We found that the EPSC paired-pulse ratio (PPR) in iDkk1 mice is higher than in control mice, consistent with a decrease in release probability (Fig. 5d). Altogether these results demonstrate that deficient Wnt signalling in the adult striatum induces structural and functional defects at cortico-striatal presynaptic terminals.

### Dkk1 triggers degeneration of dopamine synapses

**Figure 6:**
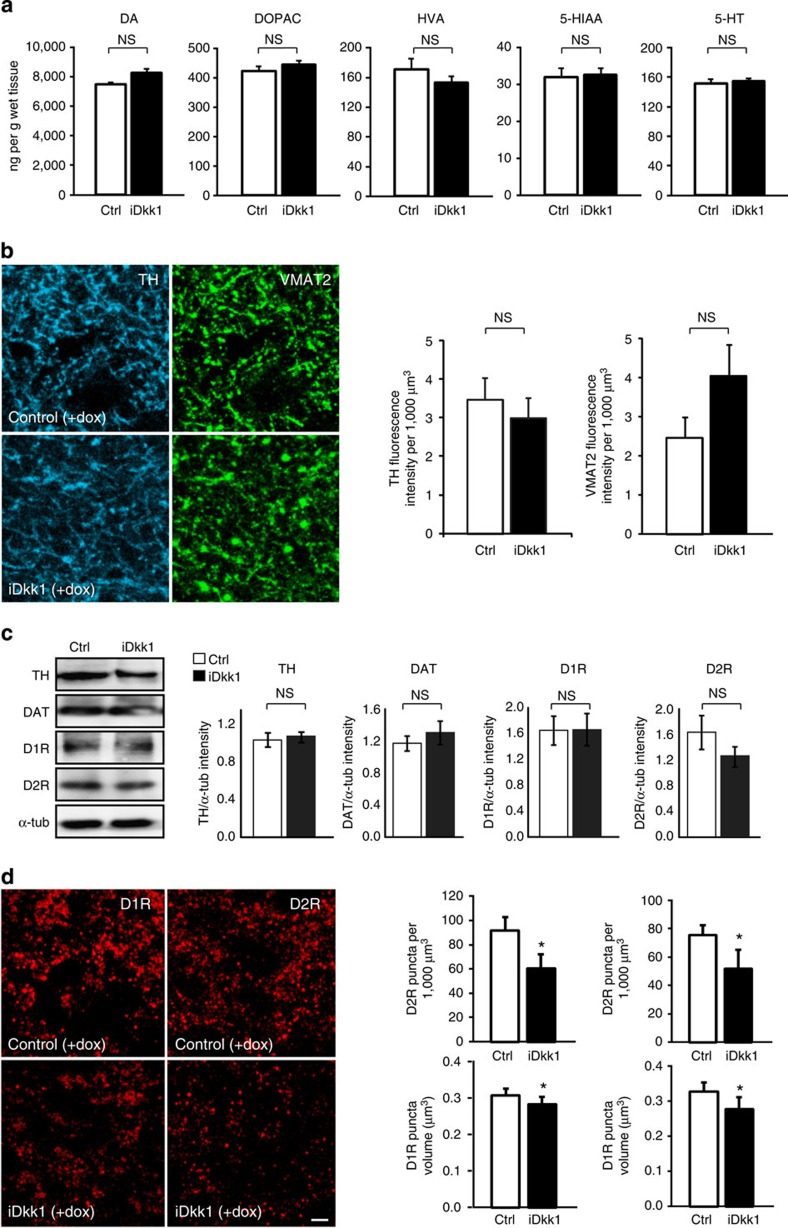
Blockade of Wnt signalling triggers the loss of D1R and D2R clusters in the dorsal striatum, without dopamine axon retraction. (**a**) Levels of dopamine (DA) and its metabolites, 3,4-dihydroxyphenylacetic acid (DOPAC) and homovanillic (HVA), as well as 5-HT and 5-HIAA, in the adult dorsal striatum. Student’s *t-*test, *n*=10 control and *n*=7 iDkk1 mice. (**b**) Left, maximum projection confocal images of striatal sections showing dopaminergic axons labelled with tyroxine hydroxilase (TH) and the vesicular monoamine transporter 2 (VMAT2). Right, quantification of total fluorescence intensity. Kruskal–Wallis ANOVA, 3 images from each slice, 3–4 slices per mouse, 6 mice per condition. Scale bar, 5 μm. (**c**) Western blot and quantification of striatal proteins. Kruskal–Wallis ANOVA, *n*=4 mice for each condition. (**d**) Left, maximum projection confocal images of striatal sections showing D1R or D2R puncta (clusters). Left, quantification of puncta. *P<0.05, one-way ANOVA with replication, 3–6 images from each mouse, and 4 mice per condition. Scale bar, 5 μm. Error bars represent s.e.m. NS, not significant.

The dopaminergic input from the substantia nigra is an important regulator of glutamatergic transmission in striatal synapses. We therefore examined the dopaminergic synapses in the dorsal striatum of iDkk1 mice. We first determined the levels of dopamine and its derivatives, 3,4-dihydroxyphenylacetic acid and homovanillic acid using high-performance liquid chromatography in the adult striatum of iDkk1 exposed to doxycycline. We found that dopamine levels are not affected (Fig. 6a). In addition, no significant changes in other neurotransmitters such as serotonin (5-HT) and its metabolite 5-hydroxyindoleacetic acid levels were detected in iDkk1 mice (Fig. 6a).

Next, we examined whether the input from dopamine cells into the striatum was altered in adult iDkk1 mice. The total intensity of tyroxine hydroxylase (TH)- or vesicular monoamine transporter 2 (VMAT2)-positive processes is unchanged (Fig. 6b), indicating that Dkk1 does not affect dopaminergic axons or the dopamine content of these axons.

To investigate possible changes in the sensitivity of MSNs to dopamine, we analysed the D1 and D2 dopamine receptors (D1R and D2R). Total levels of TH, dopamine transporter (DAT) and D1R and D2R proteins are not affected in iDkk1 mice, as determined by western blot analyses (Fig. 6c). Interestingly, blockade of Wnt signalling significantly reduces the volume and number of D1R and D2R puncta (Fig. 6d). Altogether these results suggest that deficient Wnt signalling causes the disassembly of dopamine receptor puncta without affecting the total protein levels. This effect could be due to decreased clustering and/or receptor trafficking, changes in the rate of internalization or due to dispersal of the receptors.

### Striatal synaptic degeneration impairs motor coordination

**Figure 7:**
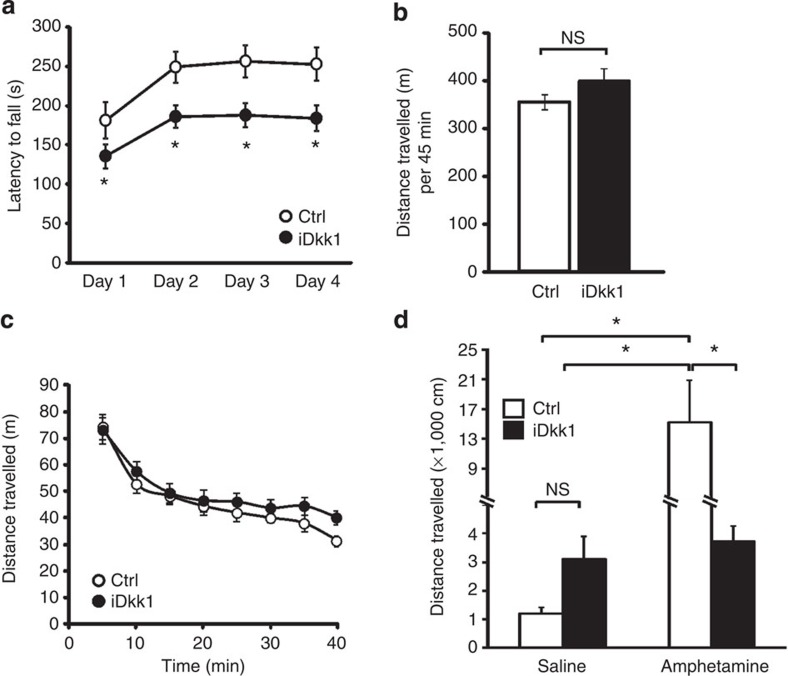
Blockade of Wnt signalling impairs striatal-mediated function. (**a**) Performance on the rotarod was evaluated as the latency to fall from the rod, in mice fed for 8–13 days with doxycycline. *n*=12 control (Ctrl) and *n*=19 iDkk1 mice, *P<0.05, ANOVA for repeated measures. (**b**) Spontaneous locomotion was measured as the total distance travelled over the 45-min period. (**c**) Spontaneous locomotion represented as distance travelled vs time in 5-min bins over a 45-min period. *n*=8 mice, Student’s *t*-test and ANOVA for repeated measures were used for data on left and right panels, respectively. (**d**) Total distance travelled recorded immediately after intraperitoneal injection of saline or amphetamine (2 mg kg^−1^) over a 60-min period. *P<0.05, two-way ANOVA, 8 animals were injected with saline (controls) or amphetamine, 9 iDkk1 mice were injected with saline and 11 with amphetamine. Error bars represent s.e.m.

Given the significant defect in the cortico-striatal glutamatergic and dopaminergic synapses in the striatum of iDkk1 mice, we evaluated whether the striatum-regulated behaviour was affected. IDkk1 mice do not exhibit defects in the open-field task indicating that general sensorimotor activity, exploratory and anxiety-like behaviour are normal (data not shown). In addition, mice move normally suggesting no defects in cerebellar function. We next examined motor coordination and learning using the rotarod, a reliable motor-learning task that provides information about dorsal striatal function during different phases of learning^38^. Indeed, disruption of dorsal striatal synaptic plasticity and function leads to impairments in motor learning in the rotarod^38^,^39^. IDkk1 mice exhibit impaired motor performance, as the latency to fall is significantly reduced compared with controls (Fig. 7a). IDkk1 and control mice exhibit a similar between-session improvement (Fig. 7a), suggesting that iDkk1 animals are able to learn. However, iDkk1 mice show motor impairment initially and after training on the rotarod (Fig. 7a), indicating that performance, but not task learning, is impaired in Dkk1-expressing mice.

### D1R loss impairs amphetamine-induced locomotion

As iDkk1 mice exhibit the loss of D1R clusters in the dorsal striatum, we evaluated whether these mice show impaired locomotion. Evaluation of non-habituated control and iDkk1 animals for a period of 45 min revealed no significant difference in total locomotion (Fig. 7b), indicating that iDkk1 mice show similar spontaneous locomotion to control littermates.

Stimulation of dopamine signalling by psychostimulants such as amphetamine or cocaine causes an increase in locomotion through D1Rs^40^,^41^,^42^,^43^. As Wnt blockade induces degeneration of D1R clusters, we predicted that the response to amphetamine would be impaired in iDkk1 mice. As previously reported, amphetamine (2 mg kg^−1^) significantly increases locomotion in control animals when compared with saline-injected mice (Fig. 7c). In contrast, amphetamine does not increase locomotion in iDkk1 mice compared with iDkk1 mice injected with saline (Fig. 7c). Although iDkk1 mice are slightly hyperactive compared with controls when injected with saline, this difference is not statistically significant (Fig. 7c). As iDkk1 mice exhibit similar dopamine, VMAT2 and DAT levels to control mice (Fig. 6a–c), the lack of response to amphetamine is likely due to defects in the stability of postsynaptic D1R clusters. These findings indicate that blockade of Wnt signalling impairs the locomotion in response to increased dopamine release.

## Discussion

Here we report a novel role for Wnt signalling in the maintenance/stability of excitatory and dopaminergic synapses in the adult striatum. We demonstrate that *in vivo* blockade of Wnt signalling by inducibly expressing the secreted Wnt antagonist Dkk1, results in a significant degeneration of dopaminergic and cortico-striatal excitatory synapses in the adult striatum and a decrease in glutamate release from cortico-striatal afferents. Importantly, these synaptic defects correlate with impairment in striatal-regulated function. Our studies shed new light into the signalling pathways that control synaptic maintenance in the adult striatum.

Induction of Dkk1 expression in the adult striatum blocks Wnt signalling without affecting cell viability. Specific induction of Dkk1 expression for a period of 2 weeks in adult iDkk1 transgenic mice show a decrease in the number of β-catenin puncta in the dorsal striatum, indicating that Dkk1 indeed blocks the canonical Wnt signalling pathway^26^,^27^. Previous studies suggest that increased Dkk1 levels may contribute to cell loss after cerebral ischaemia, epilepsy and in neurodegenerative diseases^34^,^35^,^36^. However, we have previously shown that Dkk1 does not affect cell viability in mature hippocampal neurons^21^. Consistent with this study, we demonstrate here that induction of Dkk1 expression does not induce cell loss in the adult striatum or in the somatosensory cortex (an area that innervates the striatum). Moreover, we observed neither dopaminergic axon retraction nor changes in dopamine levels and its metabolites in the striatum, suggesting that the viability of the dopaminergic neurons in the substantia nigra is unaffected by striatal expression of Dkk1. These findings strongly support our conclusion that induction of Dkk1 in the adult striatum does not trigger neuronal degeneration.

Endogenous Wnt signalling is required for synaptic integrity in the adult striatum. Although a role for Wnt signalling in synapse formation and function has been well established, its contribution to synaptic stability in the adult brain has remained unexplored. Here we demonstrate that endogenous Wnts are required for the integrity of glutamatergic cortico-striatal synapses and stability of D1R and D2R clusters in the adult striatum. Interestingly, glutamatergic inputs from the thalamus are unaffected in iDkk1 mice, suggesting that blockade of Wnt signalling induces the disassembly of specific synapses.

Blockade of canonical Wnt signalling in the adult induces presynaptic structural and functional changes at excitatory synapses. Importantly, Dkk1 mice exhibit a significant increase in PPR indicative of reduced probability of release accompanied with a reduced number of vesicles at the ultrastructural level. Thus, deficiency in Wnt signalling causes a significant disruption of cortico-striatal transmission by affecting presynaptic function.

Wnt deficiency in the striatum results in impaired locomotion characteristic of striatal dysfunction. IDkk1 mice exhibit deficits in motor coordination in the rotarod test as observed in D1R and D2R null mice or after pharmacological inhibition of these receptors^38^,^44^. Interestingly, both D1R and D2R are necessary for motor coordination during the learning stages of the task, whereas only D2R is important for motor performance once the task becomes a habit^38^. IDkk1 mice exhibit impaired motor coordination during learning and after 4 days of training, suggesting that initial impairment in coordination may be due to the decrease in both D1R and D2R clusters, whereas impaired performance observed once the task is learnt could be due to the decreased number of D2R clusters. In addition, iDkk1 mice are irresponsive to amphetamine, a psychostimulant that increases extracellular dopamine levels through blockade of dopamine uptake, increased dopamine release from intracellular stores and the reversal of the DAT^42^,^43^. This defect is likely to be due to the decreased number of D1R puncta rather than to a presynaptic defect, as neither dopaminergic axon retraction nor changes in dopamine levels are observed in the striatum of iDkk1 mice. These findings highlight the importance of endogenous Wnt signalling in dopaminergic transmission at striatal neurons.

Loss of synapses is an early event in several neurodegenerative diseases, including PD and HD^5^,^6^. Indeed, PD and HD mouse models exhibit significant deficits in glutamatergic and dopaminergic neurotransmission and motor defects before or in the absence of neuronal degeneration^8^,^9^,^16^,^17^,^18^,^19^,^20^. Similarly, iDkk1 mice exhibit deficits in glutamatergic and dopaminergic transmission that correlate with impaired striatal-mediated behaviour. Although these changes occur in the absence of neuronal degeneration, the phenotype of iDkk1 mice has striking similarities to those observed in PD and HD genetic models. Although a genetic link between dysfunction in Wnt signalling and PD has not been reported, a key component of the Wnt signalling pathway, Dishevelled (Dvl), has been recently shown to interact with LRRK2, the most prevalent and dominant mutation in PD^45^. In addition, Vps35, an essential retromer subunit that is linked to autosomal dominant late-onset PD^46^, regulates Wnt secretion^47^,^48^. These studies suggest that deficient Wnt signalling could contribute to the pathogenesis of PD during the early stages. In conclusion, the iDkk1 mice recapitulate the early stages of neurodegenerative diseases when synapses are compromised. Here, we introduce iDkk1 mice as a model system to elucidate the molecular mechanisms that lead to synaptic degeneration and dysfunction in the absence of neuronal death.

## Methods

### *In vivo* induction of Dkk1 expression in the adult striatum

All experiments were performed in accordance with the Animals Scientific procedures Act UK (1986). Heterozygous tetO-Dkk1 transgenic mice^28^ were crossed with the heterozygous CaMKIIα-rtTA mice^29^ to obtain the double-transgenic animals (iDkk1). The mice background was C57BL/6J. Genotypes were confirmed by PCR using DNA isolated from ear notches. For CaMKIIα-rtTA, the primers used were forward 5′-TGCCTTTCTCTCCACAGGTGTCC-3′, reverse 5′-GAGAGCACAGCGGAATGAC-3′; for tetO-Dkk1, forward 5′-GCGTCCTTCGGAGATGATGG-3′, reverse 5′-AAATGGCTGTGGTCAGAGGG-3′.

IDkk1 and control mice were administered food supplemented with 6 mg kg^−1^ doxycycline (Datesand group) for 14 days unless otherwise indicated. Doxycycline-fed wt and single-transgenic animals were used as controls. Experiments were performed in 3–6-month-old mice. Both male and female mice were used for cellular biology experiments. For behavioural and electrophysiological experiments only males were used.

### RT-PCR analysis of gene expression

RNA was extracted from the striatum of three P15 and three adult mice (3 months old) using Trizol (Invitrogen) and treated with DNAse I (Sigma). First, strand complementary DNA synthesis was performed with AMV Reverse Transcriptase (Promega) according to the manufacturer’s instruction. PCR was performed using GoTaq Polymerase (Promega). The primers used are indicated in Supplementary Table 1.

### *In situ* hybridization

Brains of iDkk1 or control mice were snap-frozen in isopentane cooled in liquid nitrogen and stored at −80 °C. Twelve μm sagittal sections were cut in a cryostat, air dried and fixed in 4% paraformaldehyde (PFA) in phosphate-buffered saline (PBS) prior to labelling^49^. Probes^50^ were labelled with digoxigenin using the DIG RNA labelling kit (Invitrogen) according to the manufacturer’s instructions.

### Immunohistochemistry

IDkk1 and control mice were anaesthetized, decapitated and brains rapidly dissected and fixed overnight in 4% PFA at 4 °C. The brains were next washed with PBS, immersed in 30% sucrose/PBS and frozen in isopentane or OCT in dry ice. Twenty-μm-thick cryosections were collected onto slides (Superfrost Plus–VWR) and stored at −20 °C.

TUNEL staining was performed with ApoptoTag Red In Situ Apoptosis detection kit (Chemicon International) according to the manufacturer’s instructions. Positive controls were obtained by digesting brain sections with DNAse I 1 μg ml^−1^ in 30 mM Tris buffer, 4 mM MgCl_2_, 0.1 mM dithiothreitol, pH 7.4 for 10 min at room temperature (RT).

To detect apoptosis using cleaved-caspase 3 staining, brain slices were blocked in 10% donkey serum, 0.02% Triton X-100 in PBS for ~4 h at RT and then incubated with a primary antibody anti-cleaved-caspase 3 (Cell Signaling, 1:500) overnight at 4 °C and a secondary antibody conjugated with Alexa 568 (Invitrogen) for 2 h. Finally, brain sections were incubated with Hoescht for 5 min and mounted in Fluoromount-G. As a positive control, horizontal acute brain slices of cortex from P2 mice were incubated at 37 °C for a period of 4 h and then stained as above.

For dopaminergic synapses, brain sections were blocked and incubated with primary and secondary antibodies as described above. Primary antibodies were D1R (Sigma, 1:200), D2R (Millipore, 1:500), VMAT2 (Acris, 1:1,000) and TH (Millipore, 1:1,000). Secondary antibodies conjugated with Alexa 488, Alexa 568 and Alexa 647 were from Invitrogen (dilution 1:800).

### Acute brain slices and staining of glutamatergic synapses

Brains were rapidly dissected and placed into ice-cold artificial cerebro-spinal fluid in mM: 150 NaCl, 3 KCl, 2 CaCl_2_, 1.25 NaH_2_PO_4_, 26 NaHCO_3_, 10 D-glucose, pH 7.4. Sagittal slices (200–300 μm) were cut at 4 °C with a vibratome (Campden Instruments) and fixed in 4% PFA/4% sucrose in PBS for 20–30 min at RT. Sections were subsequently washed with PBS and blocked in 10% donkey serum, 0.02% Triton X-100 in PBS for ~4 h at RT. Primary antibodies against vGlut1 (Millipore, 1:1,000), vGlut2 (Millipore, 1:1,000), PSD95 (Thermo Scientific, 1:500) and β-catenin (BD transduction labs, 1:1,000) were incubated overnight at 4 °C. Secondary antibodies were as described above. Slices were washed in PBS and mounted in Fluoromount-G (SouthernBiotech).

### Confocal microscopy

Confocal images were acquired in an Olympus FV1000, Leica SP1 or Leica DMRE confocal microscopes using a 60 × 1.35 numerical aperture (NA) oil objective or 63 × 1.40 NA oil objective for Olympus and Leica microscopes, respectively. Image stacks of 8 equidistant planes (~200 nm) of 76 nm per pixel × 76 nm per pixel were taken for each field. Analysis was performed using Volocity software (Perkin Elmer). Synaptic and β-catenin puncta volume and number as well as VMAT2 and TH volume and intensity were quantified using custom Volocity protocols with standard thresholding techniques, and punctum density was normalized to the volume of the imaged field.

To quantify synaptic puncta and synapses, we first selected puncta using a threshold over the total fluorescence intensity of the image. Selected puncta were those with a fluorescence intensity <40% of the total image fluorescence intensity (the percentage of intensity used for thresholding varied according to the fluorophore used, from 20 to 50%). Second, when two or more puncta were identified as one, they were separated into individual puncta using a custom protocol in Volocity (‘separate touching objects’) with a puncta size guide of 0.5 μm. Synapses were determined when vGlut1 or vGlut2 puncta overlapped with PSD puncta in >1 pixel. Finally, the number, the volume and the mean fluorescence intensity of the puncta were measured.

To quantify the total fluorescence intensity of TH and VMAT, axons were selected when the fluorescence intensity of TH or VMAT was >40% of the total fluorescence intensity of the image. Total and mean fluorescence intensity was quantified within these selected areas.

### Quantification of monoamines and their metabolites

The concentration of dopamine, 3,4-dihydroxyphenylacetic acid, homovanillic acid, serotonin (5-HT) and 5-hydroxyindolacetic acid (5-HIAA) were analysed from the striatum of iDkk1 and control mice, using HPLC with electrochemical detection^51^. Brain samples were homogenized in six parts of 0.2 M HClO_4_ and one part of antioxidant solution containing oxalic acid combined with acetic acid and L-cysteine. The homogenates were centrifuged at 20,800 *g* for 35 min at 4 °C. The supernatant (350 μl) was transferred to a 0.5-ml Vivaspin filter concentrator (10,000 MWCO PES; Sartorius Stedim, Goettingen, Germany) and centrifuged at 8,600 *g* for 35 min at 4 °C. Filtrate containing monoamines was analysed using a Gemini 3-μm column (C18, 110 Å, 75 × 4.6 mm; Phenomenex, USA) kept at 45 °C with a heater (Croco-Cil, Bordeaux, France). The mobile phase consisted of 0.1 M NaH_2_PO_4_ buffer, 150 mg l^−1^ of octane sulphonic acid, 5% methanol and 450 mg l^−1^ EDTA, pH 3.0 using H_3_PO_4_. Pump (ESA Model 582 Solvent Delivery Module; ESA, Chelmsford, MA) equipped with a pulse damper (SSI LP-21, Scientific Systems, State College, PA) provided a 1 ml min^−1^ flow rate. Hundred microlitres of filtrate was injected into the chromatographic system with a Shimadzu/SIL-20AC autoinjector (Shimadzu, Kyoto, Japan). Monoamines and their metabolites were detected using ESA CoulArray Electrode Array Detector, and chromatograms were processed and concentrations of monoamines calculated using CoulArray1 for windows software (ESA). Monoamine and metabolite values were calculated as nanograms per gram (ng g^−1^) wet weight of tissue.

### Golgi staining and spine quantification

Brains were removed and immersed in Golgi solution (FD Rapid GolgiStain Kit, FD Neuro Technologies) for 7 days. Impregnation steps were performed according to the manufacturer’s instructions. The specimens were frozen and cut on a Leica CM3050 cryostat. Serial sagittal sections of 100 μm thickness were collected on slides (Superfrost Plus–VWR) and left to dry in the dark at RT for 48 h. Staining was done as suggested by the manufacturer. Slides were dehydrated in graded ethanol, cleared with xylene and mounted with Permount (TAAB Laboratories). Single-plane images were captured on an Olympus BX60 wide-field microscope with a 100 × 1.3 NA oil objective and analysed with Volocity software. Spines, defined as dendritic protrusions with a definable head, were counted manually on stretches of secondary or tertiary dendrites and normalized to dendrite length.

### EM

Brains of iDkk1 or control mice fixed with 4% PFA, 0.5% glutaraldehyde in 0.1 M Milloning phosphate buffer, pH 7.4 overnight at 4 °C were cut into coronal sections (200 μm thickness) on a vibratome and postfixed in 1% osmium tetroxide in cacodylate buffer for 1 h. Slices were then stained in aqueous uranyl acetate for 45 min, dehydrated in graded alcohol and embedded in resin. Ultrathin sections (70 nm) of silver–gold interference colour were cut and collected on a 200-mesh-grid^52^. Electron micrographs were acquired using a JEOL1010 electron microscope at a magnification of × 40,000 or × 60,000 in the dorsal striatum just below the corpus callosum. Asymmetric synapses were evaluated only when a clear pre- and postsynaptic membrane was visualized, with the presence of a prominent PSD density in the postsynaptic and vesicles in the presynaptic site^53^,^54^. PSD length and synaptic cleft length were quantified by visual placement of a line tool across the maximum head (for spines) or the longest axis. Vesicles were counted within 200 nm of the active zone.

### Western blotting

Homogenates prepared from striatum of iDkk1 or control mice (*n*=4) were run on SDS–polyacrylamide gel electrophoresis, and western blots were probed with antibodies against DAT (Millipore), α-tubulin (Sigma) and the TH (1:1,000), D1R (1:500) and D2R (1:500) mentioned above. Band intensity was quantified using ImageJ software (NIH). Protein content was normalized against α-tubulin (1:1,000). Full blots can be found in Supplementary Fig. 6.

### Rotarod

Mice fed for 8–13 days were placed on a rotarod (Med associates) accelerating from 4–40 r.p.m. in 3 min and the latency to fall was recorded. Mice received five consecutive trials per session, one session per day for 4 days. Maximum trial length was 5 min, after which the animals were returned to the cage. Rest between trials was ~30 s (ref. 55).

### Spontaneous locomotion

Naive animals fed with doxycycline for 14–21 days were placed in 44 × 44-cm polycarbonate cages with opaque walls for 45 min. Locomotion was recorded from a camera placed above the cages and analysed with Daqtrack video tracking software (Axona).

### Amphetamine-induced locomotion

Motor activity was recorded in 8 identical activity monitor chambers consisting of transparent boxes (43 × 43 cm) equipped with 16 infrared light emitters and detectors (Med Associates). These were connected to a computer that counts the number of times the photobeams are broken (as the animal crosses between the emitter and the detector). The total number of horizontal beam breaks was used as a measure of locomotion. Mice fed with doxycycline for 14–21 days were individually placed in the activity chamber and habituated for 1 h prior to the experiment. After habituation, mice were administered amphetamine (D-amphetamine sulphate, Sigma, 2 mg kg^−1^ in saline) or saline intraperitoneally. Locomotor activity was monitored for 1 h immediately after the injection by registering the infrared photobeam interruptions.

### Electrophysiology

Brains were quickly removed and placed in an oxygenated ice-cold solution containing (in mM): 100 sucrose, 25 glucose, 75 NaCl, 25 NaHCO_3_, 1 CaCl_2_, 4 MgCl_2_, 2.5 KCl, 1.25 NaH_2_PO_4_.2H_2_O, 0.25 kynurenic acid, 2 pyruvic acid and 0.1 EDTA. Sagittal brain slices (300 μm) were transferred to an incubating chamber containing (in mM): 125 NaCl, 26 NaHCO_3,_ 2.5 KCl, 1.26 NaH_2_PO_4_.2H_2_O, 25 glucose, 1 MgCl_2_ and 1 CaCl_2_ oxygenated with 95% O_2_-5% CO_2_. After a 1-h recovery period, slices were visualized under the microscope with infrared Nomarski differential interference contrast optics (Axioskop FS; Zeiss). All recordings were made at RT. Whole-cell voltage clamp recordings were obtained from dorsal striatal MSNs identified by morphological and electrophysiological criteria (cell size, membrane capacitance and cell response to depolarizing current injection). The sEPSCs were recorded in the presence of 10 μM bicuculline, a selective GABA_A_ receptor antagonist. mEPSCs were recorded in the presence of 10 μM bicuculline and 100 nM TTX. Recordings were performed at −70 mV holding potential using glass pipettes, pulled from standard borosilicate glass capillaries filled with a pipette solution containing (in mM): 139 D-gluconic acid lactone, 10 HEPES, 10 EGTA, 10 NaCl, 0.5 CaCl_2_, 1 MgCl_2_, 1 ATP and 0.5 GTP adjusted to pH 7.2 with CsOH. Currents were recorded using an Axopatch 200A Amplifier, filtered at 1 kHz and digitized in the computer at 10 KHz. The data were acquired with Win EDR and analysed using Win EDR and WinWCP (freely available at http://spider.science.strath.ac.uk/sipbs/software_ses.htm).

To evaluate the PPR, MSNs afferents were electrically stimulated using a bipolar concentric stimulating electrode placed in the anterior portion of the deep layers of the motor cortex, at the level of the white matter between the cortex and the striatum. Paired-pulse stimulation was performed to record evoked synaptic currents (eEPSCs). This was performed by giving 2 consecutive pulses separated by 50 ms every 10 s up to 20 times. The ratio (PPR) of the second evoked current to the first was compared between wt and iDkk1 mice. Differences in the intensity of stimulation required to obtain a given synaptic event were not observed across genotypes (range: 8–30 V, 0.05 ms pulse duration).

### Statistical analyses

For all set of samples, normality and homogeneity of variance were confirmed by Lilliefords and *χ*^2^-tests, respectively. If samples showed a normal distribution and homogeneity of variance, parametric tests were applied as described below. Otherwise, samples were analysed by a non-parametric test.

β-catenin puncta were analysed by one-way analysis of variance (ANOVA) with blocking and replication. Mice were considered as blocks. For each mouse, 9–12 fields were imaged from 4 different slices and four mice were used for each condition. No statistical significance was observed between mice.

Synaptic puncta and colocalization of synaptic markers were analysed by one-way ANOVA with blocking and replication. The number of experiments was considered as blocks; typically three independent experiments, each of them with 1–3 mice from each condition. A minimum of 15 images were obtained from 3 different slices per mice.

TH and VMAT total fluorescence intensity were analysed by non-parametric Kruskal–Wallis ANOVA, as normality could not be confirmed for control samples. Three images were imaged and analysed per slice, 3–4 slices per mouse, six mice per condition.

The number of asymmetric synapses was analysed in EM micrographs by non-parametric Kruskal–Wallis ANOVA. Eighteen to twenty images from each mouse, 4 mice for each condition were analysed. The number of vesicles was analysed by one-way ANOVA with blocking and replication. Mice were considered as blocks. Vesicles were counted in 17–25 synapses from each control mouse and 15–19 synapses from each iDkk1 mouse, four mice were used for each condition. No statistical significance was observed between mice. For the analysis of PSD length and width and cleft width, data were pooled and analysed by non-parametric Kruskal–Wallis ANOVA. More than 140 synapses obtained from 4 different mice per condition were analysed.

Number of spines in Golgi-stained images was analysed by non-parametric Kruskal–Wallis ANOVA. Nine–50 dendrite stretches obtained from 4 different mice per condition were analysed.

The content of dopamine and other metabolites in the striatum was analysed by the Student’s *t*-test in 10 control mice and 7 iDkk1 mice.

Western blot data were analysed by non-parametric Kruskal–Wallis ANOVA in four mice for each condition.

PPR was analysed using the unpaired *t*-test, 14 cells from control and 18 cells from iDkk1 mice were evaluated. mEPSCs and sEPSCs were analysed by the Student’s *t*-test, 20 and 27 neurons were recorded from control and iDkk1 mice for mEPSCs, respectively, and, 16 and 17 neurons from control and iDkk1 mice for sEPSCs, respectively.

Spontaneous locomotion was evaluated by the Student’s *t*-test and ANOVA for repeated measures, eight mice for each condition. Amphetamine-induced locomotion was evaluated by two-way ANOVA, 8 controls with saline, 8 controls with amphetamine, 9 iDkk1 with saline and 11 iDkk1 with amphetamine. Rotarod data were evaluated by ANOVA for repeated measures, 12 control and 19 iDkk1 mice were used.

## Author contributions

S.G. and P.C.S. contributed to the design of the project and experiments, interpretation of the data and writing of the manuscript. S.G. performed all the molecular and cellular biology experiments and behavioural assays. D.M.L. performed the *in situ* hybridization and breeding and genotyping of mice. R.A. and A.G. contributed to design, performance and interpretation of electrophysiological experiments. J.K. performed the measurement of dopamine and its metabolites. R.A., J.K. and A.G. also contributed to writing of the manuscript. S.E.M. provided the tetO-Dkk1 mice and probes for *in situ* hybridization. P.C.S. and A.G. contributed with funding.

## Additional information

**How to cite this article:** Galli, S. *et al.* Deficient Wnt signalling triggers striatal synaptic degeneration and impaired motor behaviour in adult mice. *Nat. Commun.* 5:4992 doi: 10.1038/ncomms5992 (2014).

## Supplementary Material

Supplementary Figures and Table Supplementary Figures 1-6 and Supplementary Table 1
