# Supplementary material for: Deficient Wnt signalling triggers striatal synaptic degeneration and impaired motor behaviour in adult mice
Source: Nat Commun. 2014 Oct 16;5:4992. doi: 10.1038/ncomms5992 (PMC4218967; doi:10.1038/ncomms5992)
Supplement: Supplementary Figures and Table — Supplementary Figures 1-6 and Supplementary Table 1 [file ncomms5992-s1.pdf]

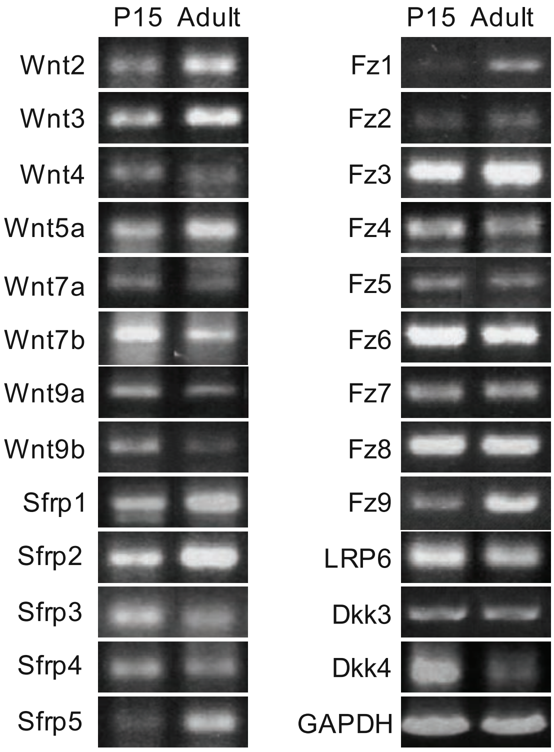

Supplementary Figure 1: Wnt signaling components are expressed in the striatum of young and adult mice. Striatum of young (P15) and adult (3 months old) mice were analyzed for mRNA expression of Wnts, Wnt receptors and secreted Wnt inhibitors by RT-PCR.

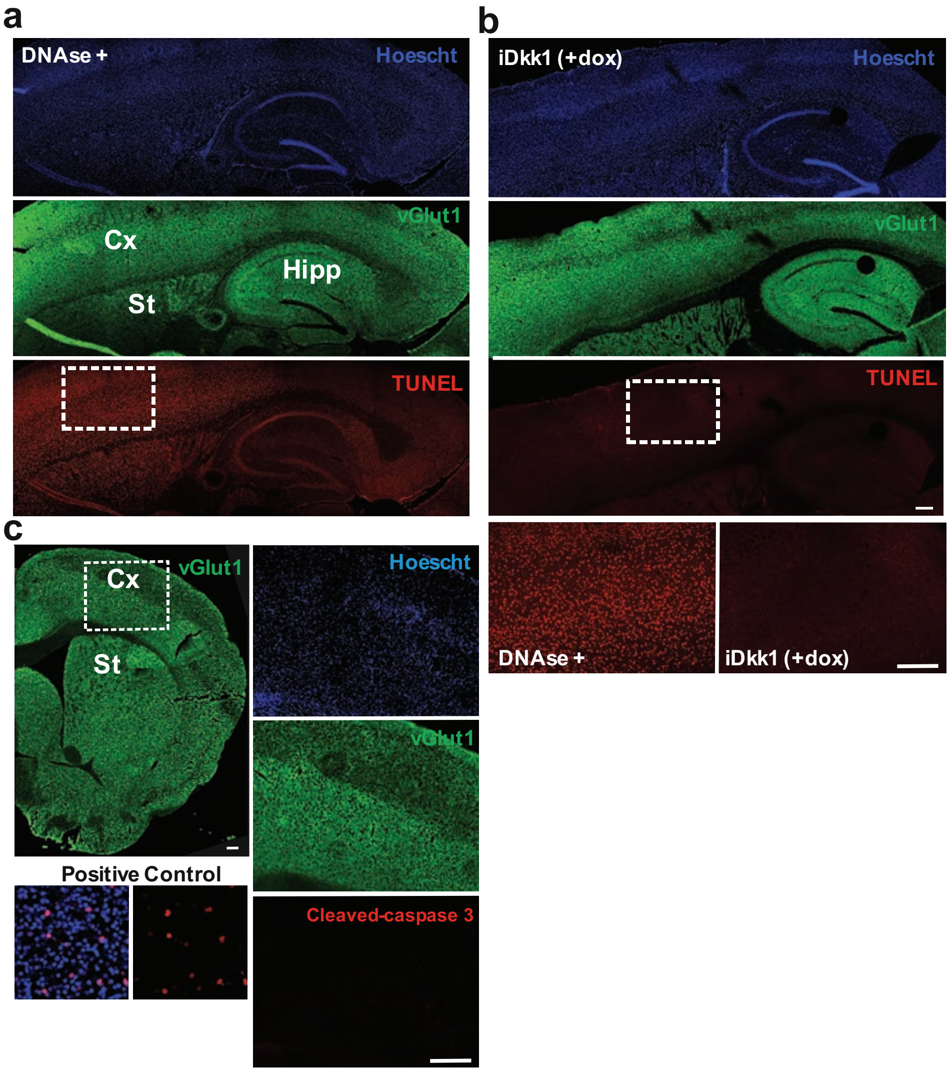

Supplementary Figure 2: Blockade of Wnt signaling does not induce cell death in the somatosensory cortex of iDkk1 mice. Apoptosis was evaluated by TUNEL assay of (a) positive control and (b) iDkk1 mice. Dorsal striatum (St), cortex (Cx) and hippocampus (Hipp) labeled with vGlut1, Hoechst and TUNEL. Brain slices treated with DNAse I were used as positive controls. Higher magnification images from areas within the broken line box are depicted below. Scale bar, 100 µm. (c) Cleaved-caspase 3 immunostaining. Top right, panoramic of coronal slice (as in figure 1d) labeled with vGlut1 depicting striatum (St) and cortex (Cx). Scale bar, 200 µm. Right, high magnification images of area indicated in the white box. Scale bar, 100 µm. Positive control, as in figure 1.

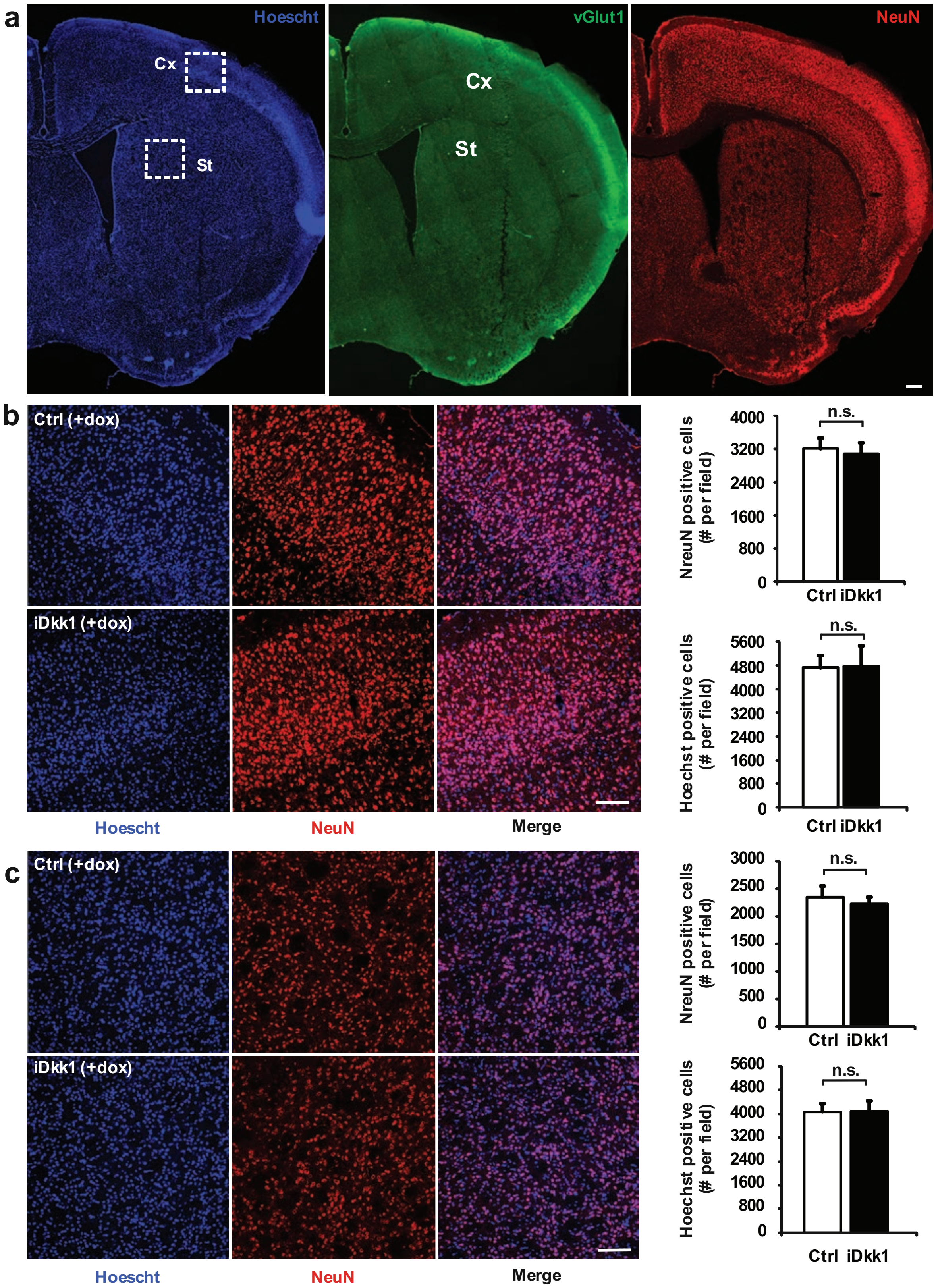

Supplementary Figure 3: Blockade of Wnt signaling does not affect neuronal viability in the striatum and cortex of iDkk1 mice. (a) Panoramic view showing the dorsal striatum (St) and cortex (Cx) labeled with vGlut1, Hoechst and NeuN. Scale bar 200 μm. Higher magnification images from somatosensory cortex (b) or striatum (c) from representative areas highlighted in the white box in (a). Quantifications are shown on the right. 1-way ANOVA with replication, 6 images were taken from 6 slices from each mouse, and 4 mice were analyzed per condition. Scale bar, 100 μm. Error bars represent s.e.m.

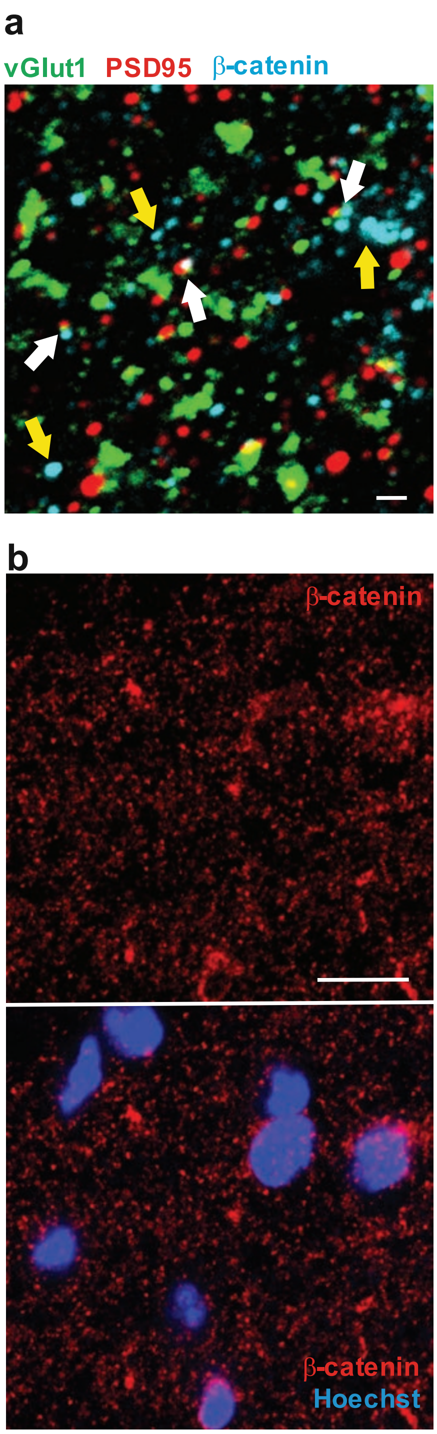

Supplementary Figure 4: β-catenin puncta localization. a) Maximum projection confocal images of dorsal striatal section showing presynaptic vGlut1, postsynaptic PSD95 and β-catenin puncta. White arrows indicate β-catenin puncta localized on cortico-striatal synapses. Yellow arrows indicate β-catenin puncta outside cortico-striatal synapses. Scale bar, 1 μm. (b) Maximum projection confocal images of lower magnification. Images show that some nuclei labeled with Hoechst contain β-catenin puncta but a large number of β-catenin puncta are in other cellular compartments. Scale bar, 10 μm.

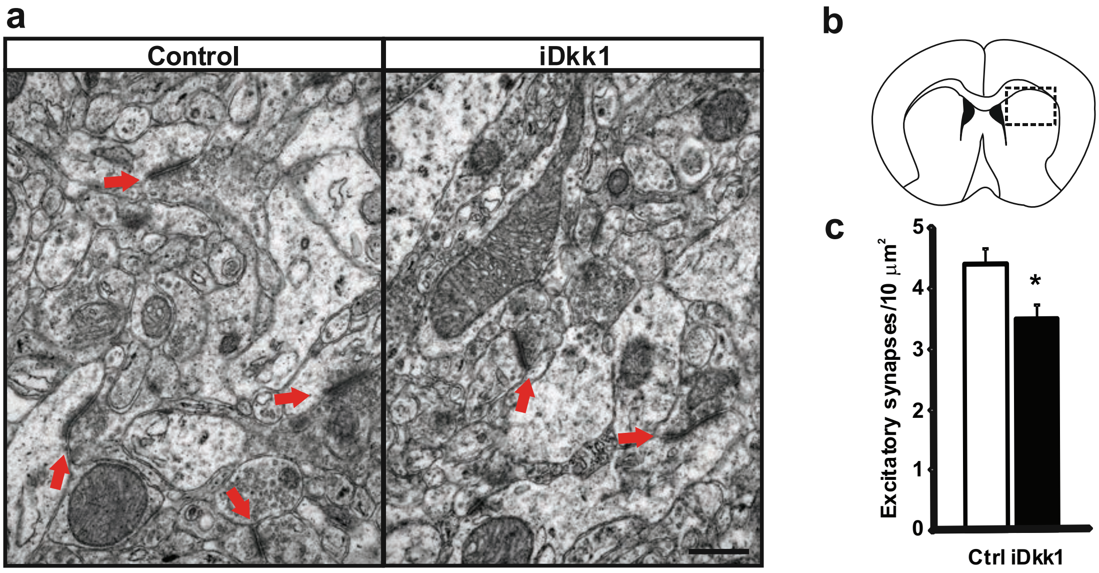

Supplementary Figure 5: Deficient Wnt signaling causes loss of glutamatergic excitatory synapses in the adult dorsal striatum. (a) Electron micrographs showing asymmetric synapses (red arrows). (b) Schematic of coronal section depicting the area examined. (c) Quantification of synapse density. Scale bar, 0.5 μm. Error bars represent s.e.m., *p<0.011, Kruskal-Wallis ANOVA, n=18-20 images from each mice, 4 mice for each condition, were analyzed.

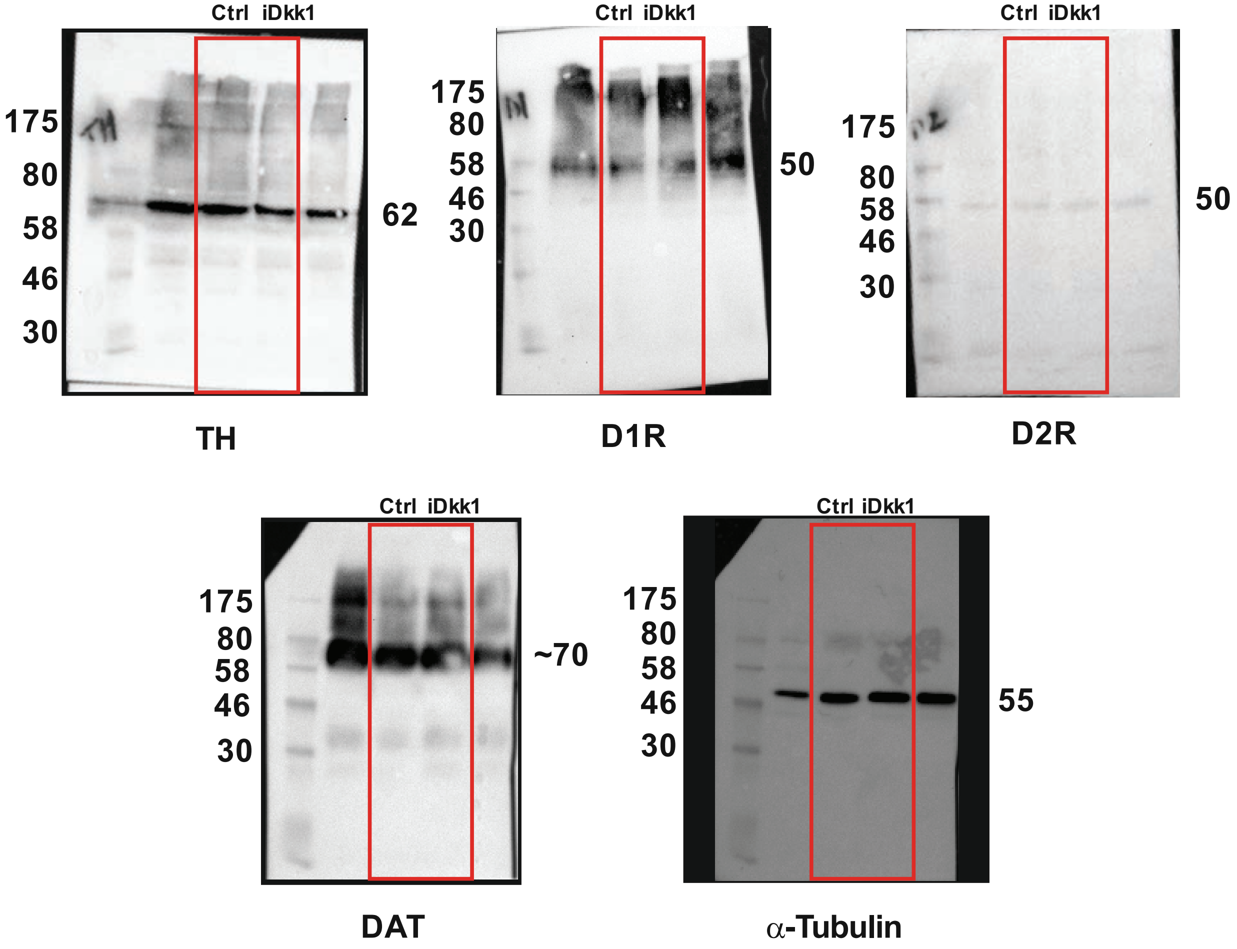

Supplementary Figure 6: Full western blots correspond to those shown in Figure 6. Molecular weight markers are indicated on the left (in kDa). Approximate molecular weight of TH, D1R, D2R, DAT and a-tubulin are displayed on the right of blots. Red box encloses bands shown in Figure 6.

Tables

Supplementary table 1: Primers used for the RT-PCR in figure S1

Gene	5’ primer	3’ primer
Wnt1	acagcaaccacagtcgtcag	Cttggaatccgtcaacaggt
Wnt2	caacagagctggaaggaag	acgaggtcatttttcgttgg
Wnt2b	cacccggactgatcttgtct	tgtttctgcactccttgcac
Wnt3	acgagaactcccccaacttt	tgggacttcgatgaatggat
Wnt3a	atggctcctctcggatacct	gggcatgatctccacgtagt
Wnt4	ctggagaagtgtggctgtga	cagcctcgttgttgtgaaga
Wnt5a	ctggcaggactttctcaagg	gtctctcggctgcctatttg
Wnt5b	tggagacaacgtggagtacg	ggcgacatcagccatcttat
Wnt6	cggtagagctctcaggatgc	attctcgaacccccagtctt
Wnt7a	ctcttcggtggtagctctgg	ctcccgactccccactttga
Wnt7b	tactacaaccaggcggaagg	gtggtccagcaagttttggt
Wnt8a	ccatcatgtacgcagtcacc	gccctgttgttgtgaaggtt
Wnt8b	gctgtgatgactcccgaaat	ttacacgtgcgtttcatggt
Wnt9a	tgctttcctctacgccatct	ttgttgtggaagtccactcg
Wnt9b	ctacgctatgacacggctgt	gtacttgctgggccggcaga
	catgagtgccagcatcagtt	aaccgcaagccttcagttta
Wnt10a	gacagtttccccacggttta	attgcttagagcccgactga
Wnt10b	caggatcccaagccaataaa	gacaggtagcgggtcttgag
Frizzled 1	ttctatgaacaggcctttcg	cctcgtgtagaacttcctcc
Frizzled 2	ggagctcctgcgctactcac	gcgctcacccagaaacttat

Frizzled 3	catgctttgaatgggccagt	ttgctgtggacactccctgc
Frizzled 4	tgacaactttcacgccgct	gtacaagccagcatcgtagcc
Frizzled 5	gtgcacagtcgtcttcctctt	gcctcgtagcgagttcaggtt
Frizzled 6	ggtcatttccaagtccatgg	ccagtgtaacatacatgtgc
Frizzled 7	ctacctagtggacatgcgtcgc	cgtagcacactccactgagtaggt
Frizzled 8	cggtgccgttgtgcaaaggc	caaccgatcgcagcgcatgc
Frizzled 9	aagacgggaggcaccaatac	aaccataactcacagcctag
Frizzled 10	ggtggccggactccctggat	gtgagcacggtgaaggcgct
Lrp 5	gatgtgcggctagtggatg	gcccgagatgacaatgttct
Lrp6	gggccgatgcaaaacttaat	cctctgttggctgaaagcat
Sfrp1	tgctcaaatgtgacaagttcc	atgagaaagttgtggctgaggt
Sfrp2	acttctcctacaagcgcagcaac	gcaggcttcacacaccttgg
Sfrp3	caagggacaccgtcaatctt	catatcccagcgcttgactt
Sfrp4	gagtggcgttcaaggatga	gactttttgggggcacttct
Sfrp5	gaccgaaagttgattggagc	gcagcagctgtccctctact
Dkk1	attccaacgcgatcaagaac	gcttggtgcatacctgacct
Dkk2	catcctcaccccacatatcc	gaggcacataacggaagcactg
Dkk3	atctcatcacctgggaactg	cttcccatcctctcccttc
Dkk4	gaagactcaagcggttctgg	gcagtcggaggttctgagac
